# Oral Health Status and Treatment Needs of Pregnant Women Attending Antenatal Clinics in KwaZulu-Natal, South Africa

**DOI:** 10.1155/2019/5475973

**Published:** 2019-03-04

**Authors:** Charlene W. J. Africa, Mervyn Turton

**Affiliations:** Department of Medical Biosciences, University of the Western Cape, Bellville, South Africa

## Abstract

During pregnancy, the oral cavity is characterised by an acidic environment and an inflammatory response brought about by vomiting and changes in hormonal levels, respectively, thereby increasing the mother's risk of developing caries. Although evidence exists to support an association between pregnancy-associated periodontal disease and adverse pregnancy outcomes, there is a paucity of studies which focus on the caries prevalence and other oral manifestations of pregnant women. The aim of this study was to assess the oral health status and treatment needs of pregnant women attending antenatal clinics in KwaZulu-Natal, South Africa. Randomly selected mothers (*n*=443) attending a maternal obstetrics unit participated in the study. A questionnaire elicited demographic information about the participants, while the measurement of decayed, missing, and filled indices (DMFT) determined their caries status. Oral lesions were noted if present. Descriptive statistics for independent variables described frequencies in the various categories of race, location, pregnancy stage, etc., with the association between 2 independent variables tested by chi-square. Dependent variables such as DMFT were expressed as means and standard deviations, and ANOVA was used to examine whether independent variables significantly influenced the DMFT. The mean DMFT was 7.18 (±4.22) with significant correlations observed between DMFT, D, M, and age. F scores differed significantly between races, location, and educational levels and showed a significant correlation with pregnancy stage. Pregnancy epulis was diagnosed in 38 (8.5%), oral lesions in 65 (14.7%), and tooth mobility in 26 (5.9%) mothers. Early oral health screening during pregnancy can ensure the overall well-being of both the mother and the foetus.

## 1. Introduction

The World Health Organization in the preamble to its constitution [1] defines health as not only an absence of disease, but as a holistic state incorporating biological, mental, and social well-being. What may be considered as normal to some may not necessarily be so for others, particularly in regions where cultural practices dictate disease experience [2].

The impact of oral health on life quality is in relation to sociodemographic factors, age group, and social class background, all of which influence education and access to health care [3]. In some cases, it is due to a general lack of understanding of the importance of oral health care as well as access to proper nutrition and medication [4].

Hormonal changes during pregnancy, along with gastric acid exposure during recurrent morning sickness, result in increased acidity in the oral cavity that can erode dental enamel [5, 6]. Furthermore, progesterone decreases plasma bicarbonate levels, thus contributing to the reduced pH [7]. This, coupled with increased sugar consumption due to dietary cravings, increases the risk for *Candida* colonisation [8] and dental caries [9–12] which, if left untreated, can lead to oral lesions, epulis, and tooth mobility [13]. It has been suggested that children born of mothers who have high caries levels are more likely to develop early caries [14, 15].

Although there is increasing evidence to support an association between pregnancy-associated periodontal disease and negative pregnancy outcomes [16], fewer studies have reported on the caries prevalence and other oral treatment needs of pregnant women. Maintaining oral health during pregnancy may be achieved by early screening and referring pregnant mothers to oral health care practitioners for treatment to complement the overall well-being of the mother and subsequently, the foetus.

## 2. Aim and Objectives

The aim of this study was to assess the general oral health status and treatment needs of pregnant women attending antenatal clinics in the province of KwaZulu-Natal (KZN), South Africa.

## 3. Materials and Methods

### 3.1. Recruitment of Participants and Ethical Considerations

The study population consisted of 443 pregnant women attending maternal obstetric units (MOU) in KZN. MOUs located at Community Health Centres (CHCs) and regional hospitals in KZN were randomly selected. Nonprobability sampling was employed to select the sites where participants were enrolled using convenience sampling.

Ethical considerations were governed by the Declaration of Helsinki [17]. The study was ethically approved by the research ethics committee of the University of the Western Cape and the Provincial Department of Health, along with the Municipal Health District managers who granted permission for the research to be conducted at the various hospitals and clinics selected.

Women attending the clinics were approached and offered the opportunity to participate in the study with the assurance that their information would remain confidential and that they would receive feedback regarding any treatment needs which may be indicated from the examination [17]. Once the details of the study were explained to them (in their mother tongue if needed), consent for participation was provided by signing a consent form with the understanding that they could withdraw from the study if and when they so desired. Those who were unable to read or write indicated their consent to participate by inserting an “X” on the consent form.

The study included patients with confirmed pregnancy aged 18 years and older and excluded mothers with chronic conditions such as diabetes mellitus [18], congenital heart disease [19], pulmonary hypertension [20], and asthma [21] and those on substance use including alcohol and illicit drugs such as cannabis, cocaine, amphetamines, and opioids [22], all of which have been associated with an increased risk of adverse pregnancy outcomes and may have confounded the outcomes of this study. Such risk groups are usually referred to specialised centres where they receive appropriate assessment and treatment during pregnancy.

### 3.2. Sample Size

The sampling of the participants from the MOUs was by means of convenience sampling, and a sample size of 400 participants was considered adequate, given a 95% confidence interval and a standard deviation of 10 (estimates of the variance (*σ*^2^ = ±5), derived from similar studies).

### 3.3. Data Collection

This cross-sectional study collected data using a standardized format which included a semistructured interview with the health professional and an administered questionnaire to collect data. The questionnaire elicited demographic information about the participants and included age at last birthday, stage of pregnancy, race, educational level (important to assess knowledge pertaining to oral hygiene status), medical history (to exclude participants with conditions which may confound the outcomes of the study), and urban or rural location (to assess local environmental conditions and availability of services).

### 3.4. Clinical Examination

Maternal oral health status was determined by recording of decayed, missing, and filled teeth (DMFT) according to WHO criteria [23] and by noting any oral lesions present.

As a part of assessing the overall health status, clinical indices to assess periodontal health status (i.e., gingival index, periodontal pocket depth, and clinical attachment level) were also recorded as published in a previous paper [24] and will not be repeated here.

### 3.5. Intraexaminer Reliability

All measurements were performed by the same dentist, and intraexaminer reliability was confirmed using Kappa statistic with 95% agreement on criteria.

### 3.6. Data Analysis

SPSS was used to perform the statistical analyses. Analyses included simple descriptive statistics in the form of frequency distributions and means. Descriptive analyses for categorical (independent) variables were expressed as percentages, mean, and standard deviation. The response rate was very high, and inferential analysis was used to generalize the results obtained from the random (probability) sample back to the population from which the sample was drawn. The univariate, chi-square test was conducted to investigate relationships between the ordinal (dependent) variable and DMFT while a bivariate ANOVA test was used to determine the correlation between DMFT scores and race, pregnancy stage, location, and educational levels. Statistical significance was defined as *p* < 0.05.

## 4. Results

Although 488 mothers were recruited to participate in the study and completed the questionnaires, 45 were excluded from the final analysis due to the exclusion criteria, incomplete data, and incoherent data. The final sample consisted of 443 mothers.

The mean age was 24.1 years (±5.3 years) with a median of 23 and a range of 18–42 years.

**Figure 1:**
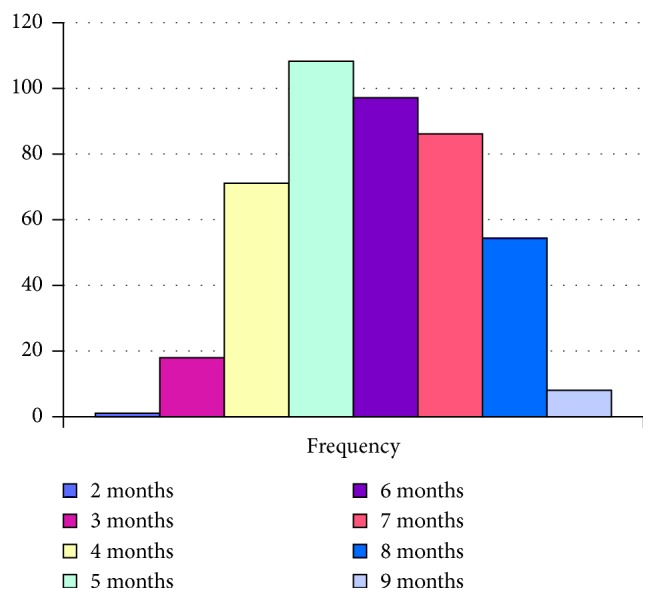
Gestational stage distribution of the study population.

Table 1 lists information regarding location, educational level, and race gained from the questionnaire while Figure 1 demonstrates the pregnancy stage distribution of the study population. Using the racial classification system to assess national and regional inequities in South Africa, the participants were classified as African (black ethnic origin), Coloured (mixed race), Indian, and White. The sample consisted predominantly of individuals of black ethnic origin (81.26%) who constitute the majority of public health facility users in South Africa, followed by Coloured (12.64%), Indian (23%), and White (4%) (Table 1).

No significant correlations were observed between pregnancy stage and regional distribution, between pregnancy stage and educational levels, and between pregnancy stage and race of the participants (*p* > 0.05).

### 4.1. Oral Health Assessment

**Figure 2:**
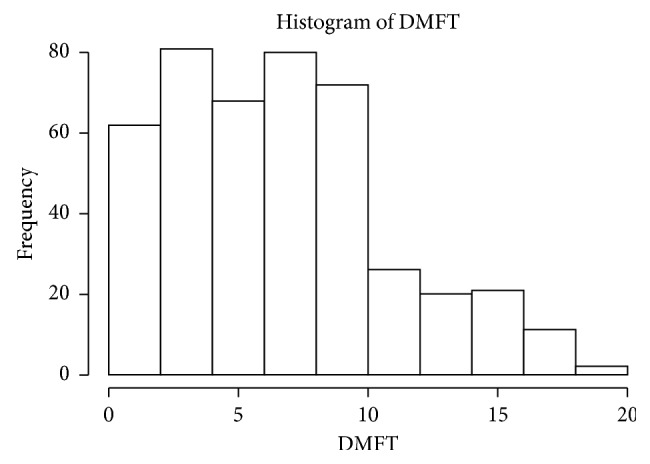
Frequency distribution of DMFT.

The frequency distribution of DMFT scores is shown in Figure 2. The mean (SD) DMFT was 7.18 (±4.22).

Correlation coefficients demonstrated significant correlations between DMFT and patient age and pregnancy stage as well as between D, M, and age and between pregnancy stage and F (Table 2). No correlation was observed between pregnancy stage and DMFT, D, and M, while F showed a significant correlation (Table 2).

Using the parametric one way ANOVA, DMFT scores differed significantly between races and educational levels (Table 3), while F scores differed significantly between races, location, and educational levels. No significant differences were observed in DMFT scores when compared with pregnancy stage or when urban and rural areas were compared.

Pregnancy epulis was diagnosed in 38 (8.5%) subjects. Oral lesions were found in 65 (14.7%) subjects of whom 46 (10.4%) presented with 1 oral mucosal lesion and 19 (4.3%) presented with 2 lesions each, diagnosed as candidiasis and/or aphthous ulcers.

Tooth mobility was recorded in 26 (5.9%) mothers, of whom 16 had severely mobile teeth in more than one quadrant.

## 5. Discussion

It has been suggested that the oral microbiome may influence the constitution of the intrauterine biome [25], thereby influencing pregnancy outcomes. We assessed the caries status and treatment needs of 433 pregnant women who attended antenatal clinics in the KZN province in South Africa, using WHO dentition status and treatment needs [22].

The mean age of the study population was representative of a substantial portion of the most economically active individuals [26, 27] and an age wherein mothers are more open to changing their attitudes to ensure better health for themselves and their babies [28, 29].

The majority of these mothers were of African race, largely disadvantaged, with many living under subeconomic conditions and very few having acquired tertiary education. Educational level is thought to determine the level of empowerment and employability of an individual, though other market factors may also contribute to determining employment status and income [26, 27], all of which contribute to access to health care [30] and disease development [3]. For that reason, many do not receive adequate health care and remain ignorant of the need to seek dental help during pregnancy.

With a mean DMFT score of 7.18, the results of this study were not unlike that reported in an Italian study [31], but higher than the score of 4.08 reported from a rural teaching hospital in India [32] and lower than the score of 12.57 reported from southeast Hungary [33] and the scores of 18 and 14 reported from Finland [34] and Brazil [35], respectively.

No significant correlation was found between DMFT and pregnancy stage, unlike earlier studies which showed that DMFT increased significantly in the 3^rd^ trimester of pregnancy compared to the first trimester [36, 37]. In this study, D and M correlated with age as reported in previous studies [33, 36, 37]. The significant correlation between F and pregnancy stage, race, location, and educational levels may be attributed to socioeconomic status and therefore treatment affordability [3, 38, 39].

Besides caries, pregnancy may also bring about other changes in the oral cavity including gingival hyperplasia and pregnancy epulis [40]. Pregnancy epulis (also known as pregnancy oral tumour, epulis gravidarum, or pyogenic granuloma), oral mucosal candidiasis, and aphthous ulcers were also observed in this study population.

Similar reports state that pregnancy epulis presents most commonly towards the end of the first trimester of pregnancy and typically recedes after delivery, occurring in approximately 0.2% to 9.6% of pregnancies [41, 42]. It is most often seen in the gingiva particularly as a result of poor oral hygiene [43] and is associated with increased progesterone levels in combination with bacteria and other local irritants [11, 44]. Such lesions are generally not treated unless they bleed, interfere with mastication, or remain and do not resolve after delivery.

Candidiasis during pregnancy may be favoured by the oral acidic environment created by the biochemical and hormonal changes which, in turn, bring about a change in the normal microbiota of the mouth, allowing for the yeasts to grow. Although caries activity has largely been attributed to the presence of oral streptococci such as *Streptococcus mutans* and *S. sobrinus,* recent studies have reported similar virulence factors expressed by *Candida albicans* [45], with oral levels of *Candida* recognised as indicators of caries activity [46] and possible vertical transmission from mothers to early caries-affected children [47]. However, the precise mutualistic and synergistic mechanisms of the interaction between *Candida* and the oral streptococci implicated in caries are yet to be determined. A recent comparison of pregnant and nonpregnant women showed that increased yeast load did not necessarily indicate candidiasis and that although yeasts were more prevalent in women who were pregnant, continuing throughout pregnancy, nonpregnant women carried a higher yeast load [8].

The actual aetiology of aphthous ulceration is not known, although it was found to be associated with vitamin and mineral deficiencies [48] and has also been observed in nonpregnant women with its occurrence seen to reduce during pregnancy. With many of these women coming from disadvantaged backgrounds, diet deficiencies would not be uncommon, thus explaining the presence of these lesions.

The tooth mobility observed in this study may be associated with the presence of relaxin, a hormone known to assist with the preparation of the birth canal for delivery and thought to relax the periodontal fibres which hold the tooth in position [6].

It is imperative for women to be screened for oral health early in pregnancy and advised on the importance of good oral hygiene, the need for regular dental visits, and changes which may be expected in the oral cavity during pregnancy [49] in order to understand what these changes represent and be reassured of the safety of treatment.

The small sample size of White women compared with other race groups may be viewed as a limitation. However, this can be explained by the fact that the clinics were situated in areas of lower socioeconomic standards and therefore a distinct reflection of the specific race groups that would be housed there, given the history of the country. Another limitation was the differences observed between self-declared medical history and that taken from the patient's records. This could account for women originally being recruited to the study and later excluded based on the physician's records which were regarded as being more accurate.

In an attempt to develop a standard method for measuring and comparing oral health data from different countries, the World Dental Foundation, Fédération Dentaire Internationale (FDI) developed the Oral Health Observatory (OHO) [50] to obtain information on oral health from individual dental practices as well as from national oral health care systems to determine how they relate to the quality of life of individuals. An OHO proof of concept study project conducted in the Netherlands to collect data on oral health and oral care in dental practices using both patient and dentist questionnaires on tablet computers [51] concluded that although collecting real-time electronic data by means of an app has the advantages of being done relatively quickly with the information sent directly to a central database, this application does not come without challenges. Such an application would be of limited value in many countries in Africa, particularly in rural areas where resources are limited.

An effective model for assessing and conceptualising the management of dental needs of pregnant women should be aimed at prevention rather than curative procedures which may sometimes impact on both the mother and her unborn baby [37, 40]. Barriers which have been identified in achieving this objective include lack of knowledge of the need for adequate oral hygiene and negative experiences with, and attitudes towards, oral health professionals [52] along with negative attitudes of dental staff towards pregnant women [53]. That said, there is no need to defer dental treatment during pregnancy since treatment after foetal organogenesis (2^nd^-3^rd^ trimester) was shown to be relatively safe [54] and should include better collaboration between patient, dental practitioners, and medical personnel with improved referral strategies [55].

Such collaboration has proved to be highly beneficial in some countries. The maternal oral screening tool (MOS) developed by George et al. [56] allows for midwives/sisters to assess the oral health risk for pregnant women by simply reporting dental health problems during pregnancy and the frequency of dental visits in the preceding 12 months. A patient with a score of ≥1 would indicate risk and referral to a dentist.

The application of screening tests such as those proposed by George et al. [56] and Africa et al. [57] along with the multifactorial model for oral health-related quality of life (OHRQoL) of Sischo and Broder [2] may contribute to achieving this collaboration between health care workers and thereby ensure the overall well-being of both mother and foetus to reduce the risk for adverse pregnancy outcomes.

## 6. Conclusion

Maintaining good oral hygiene before and during pregnancy is crucial for ensuring oral health. The study highlighted the oral health status of pregnant women within the current public health system in KZN, South Africa, and makes it imperative for the local Department of Health to make every attempt to facilitate an improvement in oral health and quality of life through assessment, education, and proper treatment planning.

## Figures and Tables

**Table 1:** Regional distribution, educational levels, and race of participants.

Variable	Frequency (%)
Regional distribution	
Urban	403 (90.9%)
Rural	40 (9.03%)

Educational levels	
Primary	30 (6.7%)
Secondary	314 (70.8%)
Tertiary	99 (22.3%)

Race	
African	360 (81.3%)
Indian	23 (5.2%)
Coloured	56 (12.6%)
White	4 (<1%)

**Table 2:** Correlation of DMFT scores with age and pregnancy stage.

	Age		Pregnancy stage	
	*r*	*p*	*r*	*p*
DMFT	0.220	<0.001	0.057	0.232
D	0.107	0.025	0.039	0.410
M	0.200	<0.001	0.012	0.800
F	0.091	0.058	0	0.0005

**Table 3:** ANOVA comparison of DMFT with race, location, and education.

	DMFT	D	M	F
Race				
African (*n*=360)	6.889	2.938	2.825	1.155
Coloured (*n*=56)	8.661	3.125	3.482	2.160
Indian (*n*=23)	7.783	2.956	2.695	2.043
White (*n*=4)	10.00	2.000	3.000	5.000
*F*	3.681	0.263	1.186	11.59
*df*	3,399	3,438	3,439	3,439
*p*	0.012	0.852	0.314	0.0001

Location				
Urban (*n*=403)	7.15	2.92	2.851	1.419
Rural (*n*=40)	7.50	3.3	3.425	0.800
*F*	5.591	0.898	1.94	3.975
*df*	2,440	47,52	1,441	1,441
*p*	0.633	0.373	0.164	0.046

Education				
Primary	6.933	3.200	2.733	1.033
Secondary	6.822	2.907	2.780	1.178
Tertiary	8.424	3.030	3.343	2.050
*F*	5.591	0.226	2.012	8.901
*df*	2.440	2,439	2,440	2,440
*p*	0.004	0.797	0.135	0.0002

## Data Availability

The data used to support the findings of this study are available from the corresponding author upon request.
